# The MUK five protocol: a phase II randomised, controlled, parallel group, multi-centre trial of carfilzomib, cyclophosphamide and dexamethasone (CCD) vs. cyclophosphamide, bortezomib (Velcade) and dexamethasone (CVD) for first relapse and primary refractory multiple myeloma

**DOI:** 10.1186/s12878-016-0053-9

**Published:** 2016-05-17

**Authors:** Sarah Brown, Samantha Hinsley, Mónica Ballesteros, Sue Bourne, Paul McGarry, Debbie Sherratt, Louise Flanagan, Walter Gregory, Jamie Cavenagh, Roger Owen, Cathy Williams, Martin Kaiser, Eric Low, Kwee Yong, On behalf of the Myeloma UK Clinical Trials Network

**Affiliations:** Clinical Trials Research Unit, Leeds Institute of Clinical Trials Research, University of Leeds, Leeds, UK; Methodology of Biomedical Research and Public Health Programme, Universitat Autònoma de Barcelona, Barcelona, Spain; Department of Haematology, St Bartholomew’s Hospital, London, UK; HMDS Laboratory, St James’s University Hospital, Leeds, UK; Centre for Clinical Haematology, Nottingham University Hospitals, Nottingham, UK; The Institute of Cancer Research, Sutton, UK; Myeloma UK, Edinburgh, UK; UCL Cancer Institute, 72 Huntley Street, WC1E 6BT London, UK

**Keywords:** First relapse multiple myeloma, Primary refractory multiple myeloma, Carfilzomib, Phase II

## Abstract

**Background:**

Multiple myeloma is a plasma cell tumour with an annual incidence in the UK of approximately 40–50 per million i.e. about 4500 new cases per annum. The triple combination cyclophosphamide, bortezomib (Velcade®) and dexamethasone (CVD) is an effective regimen at relapse and has emerged in recent years as the standard therapy at first relapse in the UK. Carfilzomib has good activity as a single agent in the relapsed setting, and it is expected that efficacy will be improved when used in combination with dexamethasone and cyclophosphamide.

**Methods:**

MUK Five is a phase II open label, randomised, controlled, parallel group, multi-centre trial that will compare the activity of carfilzomib, cyclophosphamide and dexamethasone (CCD) with that of CVD, given over an equivalent treatment period (24 weeks), in participants with multiple myeloma at first relapse, or refractory to no more than 1 line of treatment. In addition, the study also aims to assess the utility of a maintenance schedule of carfilzomib in these participants. The primary objective of the trial is to assess whether CCD provides non-inferior activity in terms of ≥ VGPR rates at 24 weeks, and whether the addition of maintenance treatment with carfilzomib to CCD provides superior activity in terms of progression-free survival, as compared to CCD with no maintenance. Secondary objectives include comparing toxicity profiles, further summarizing and comparing the activity of the different treatment arms and analysis of the effect of each treatment arm on minimal residual disease status.

**Discussion:**

The development of carfilzomib offers the opportunity to further explore the anti-tumour efficacy of proteasome inhibition and, based on the available evidence, it is important and timely to obtain data on the activity, toxicity and tolerability of this drug. In contrast to ongoing phase III trials, this phase II trial has a unique subset of participants diagnosed with multiple myeloma at first relapse or refractory to no more than 1 line of treatment and will also evaluate the utility of maintenance with carfilzomib for up to 18 months and investigate minimal residual disease status to provide information on depth of response and the prognostic impact thereof.

**Trial registration:**

The trial is registered under ISRCTN17354232, December 2012.

## Background

Multiple myeloma (MM) is a plasma cell tumour with an annual incidence in the UK of approximately 40–50 per million i.e. about 4500 new cases per annum [1]. For younger fitter patients the current standard of care is induction therapy typically using a novel agent-based regimen consolidated with high-dose melphalan and peripheral blood stem cell rescue (termed autologous stem cell transplantation, ASCT). For older less fit patients, frontline regimens include a novel agent along with steroids and an alkylating agent, but without consolidation with ASCT. With these regimens, the majority of patients will enter a plateau phase lasting some 3–5 years, however patients will relapse and require further anti-myeloma therapy.

Current standard treatment at first relapse in the UK is the use of bortezomib (Velcade®), commonly with dexamethasone [2]. Increasingly, a third agent is added, either an alkylating agent such as cyclophosphamide (CVD), an anthracycline, doxorubicin (PAD) or thalidomide. The triple combination CVD is an effective regimen at relapse, producing response rates of up to 70 % [3–6] and has emerged in recent years as the standard therapy at first relapse in the UK, with dose adjustments tailored to age and performance status. Up to 8 cycles are administered, although many patients have their treatment withdrawn before completing 8 cycles as a consequence of neurotoxicity, more frequently seen with the intravenous (IV) mode of delivery of bortezomib. Recently, a phase 3 study comparing intravenous (IV) with subcutaneous (SC) mode of delivery has reported equivalent efficacy with significantly reduced neurotoxicity [7]. The publication of these results has led to widespread changeover from the IV to the SC use of bortezomib.

The development of carfilzomib, an irreversible epoxyketone inhibitor of the proteasome, offers the opportunity to further explore the anti-tumour efficacy of proteasome inhibition, particularly as some patients have disease that does not respond to bortezomib, or develop resistance after initial response. Phase I and II studies have shown carfilzomib monotherapy can be safely administered and produces encouraging response rates. Similarly, when given in combination with lenalidomide and low dose dexamethasone, carfilzomib is well tolerated, and there are no significant overlapping toxicities [8–13].

The Myeloma UK (MUK) study, MUK five, has been developed to further explore carfilzomib combination therapy in the relapsed or primary refractory setting. Experience with bortezomib confirms improved efficacy with good tolerability when used in combination with dexamethasone and cyclophosphamide (CVD). Carfilzomib has good activity as a single agent in the relapsed setting, and it is expected that efficacy will be improved when used in combination with dexamethasone and a third agent. Hence it is important and timely to obtain data on the activity, toxicity and tolerability of Carfilzomib in combination with cyclophosphamide and dexamethasone (CCD) in the first relapse setting. This triplet regimen has recently been reported to be a well tolerated and active regimen in the frontline setting in older patients not suitable for ASCT [14]. The study has been developed through the Myeloma UK (MUK) Early Phase Clinical Trials Network, an innovative collaboration which brings together clinical specialists and researchers, the pharmaceutical industry and NHS regulatory bodies to conduct a prioritised and strategic portfolio of myeloma clinical trials.

## Methods

### Study aims

This study will compare the activity of CCD with that of the current standard therapy at relapse, CVD, given over an equivalent treatment period (24 weeks), in participants with multiple myeloma at first relapse, or refractory to no more than 1 line of treatment. In addition, the study also aims to assess the utility of a maintenance schedule of carfilzomib in these participants.

### Primary objective

To assess whether CCD provides non-inferior activity with regard to the short-term outcome measure of ≥ VGPR rates at 24 weeks, and whether the addition of maintenance treatment with Carfilzomib to CCD provides superior activity in terms of the longer-term outcome measure of progression-free survival (PFS), as compared to CCD with no maintenance.

### Secondary objectives

- To compare the toxicity profile of CCD with that of CVD, overall and specifically with respect to peripheral neuropathy
- To explore the non-inferiority of CCD without maintenance as compared to CVD for longer-term outcome measure of PFS
- To further summarise the activity of CCD as compared to CVD with regard to:

- Complete response at 24 weeks
- Overall response at 24 weeks and within 12 months
- Maximum response within 12 months
- Maximum response overall
- Time to maximum response
- Duration of response
- Overall survival
- Time to next treatment

- To determine the proportion of participants with a negative minimal residual disease (MRD) status at the end of initial treatment phase (24 weeks of treatment) in both the CVD and CCD arms and to correlate MRD-negativity at the end of the initial treatment phase (24 weeks) with PFS for all participants
- To assess the effect of maintenance Carfilzomib on MRD status at 6 months and at 12 months post second randomisation
- To correlate treatment outcomes (Complete response CR, overall response rate ORR) and PFS with genetic subgroups
- To summarise treatment compliance.

### Study design

**Fig. 1:**
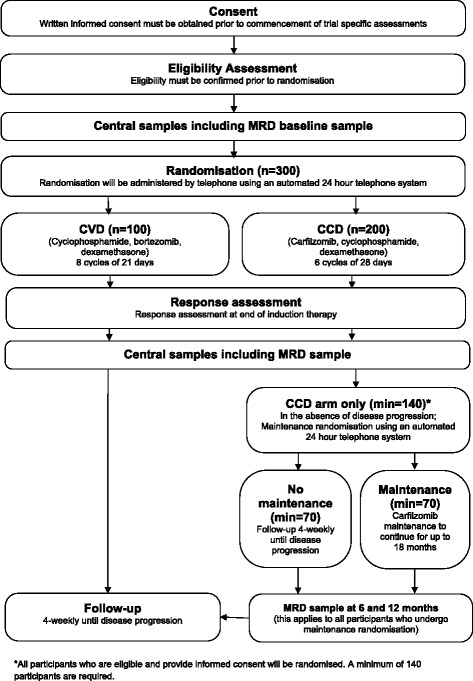
Trial flow diagram. *All participants who are eligible and provide informed consent will be randomised. A minimum of 140 participants are required

The MUK five trial is designed as a phase II randomised, controlled, parallel group, multi-centre clinical trial for participants with symptomatic myeloma at first relapse, or refractory to not more than 1 line of therapy. The trial has received national research ethics approval from the NHS National Research Ethics Service London, Fulham (REC Number: 12/LO/1078). A total of 300 participants will be randomised on a 2:1 basis to either 6 cycles of CCD or 8 cycles of CVD (both equivalent to 24 weeks of therapy), with follow-up to disease progression. Participants in the CCD arm who, at the end of the initial 6 cycles of CCD do not have evidence of disease progression, will be randomised to receive maintenance therapy with Carfilzomib or to receive no further treatment. Participants in the CVD arm will not receive maintenance therapy (Fig. 1). In order to compare the regimens with regard to activity, the trial has been designed to incorporate two co-primary endpoints: response and progression-free survival. This allows the trial to assess the activity of the two regimens within a fixed period of 24 weeks of treatment, i.e. not incorporating the maintenance phase in the CCD arm, and to compare the activity of the whole CCD regimen with and without maintenance therapy, and the whole CCD regimen without maintenance with the CVD regimen by evaluating the longer term endpoint of PFS.

### Sample size

The sample size was calculated according to each of the co-primary endpoints

- ≥ Very good partial response (VGPR)

The primary endpoint analysis is based on a non-inferiority comparison of CCD vs. CVD. Clinical discussion taking into account recent data anticipates a ≥ VGPR rate of approximately 30–40 % with CVD at first relapse [4, 15, 16]. Considerable discussion was given to whether this comparison should be performed as a superiority or non-inferiority comparison, since CCD is expected to improve ≥ VGPR rates by up to 10 %. In this setting, however, a phase II superiority trial to detect this small improvement would likely be unfeasible. A non-inferiority design is therefore used, assuming a ≥ VGPR rate of 35 % with CVD, and a ≥ VGPR rate of 45 % with CCD, with a non-inferiority margin of −5 %. Randomising participants on a 2:1 basis in favour of CCD, a total of 291 participants (CCD = 194, CVD = 97) are required to exclude a difference of −5 % from the 90 % confidence interval with 80 % power (1-sided 5 % significance level).

- Progression-free survival

The primary PFS comparison will be focused on those participants undergoing maintenance randomisation in the CCD arm, to compare maintenance with carfilzomib vs. no maintenance. It is assumed that median PFS with CVD with no maintenance will be approximately 14 months from the time of initial treatment [17]. It is hypothesised that a similar median PFS will be observed with CCD therapy with no maintenance. It is also anticipated that approximately 80 % of participants randomised to receive CCD therapy will be progression-free at the end of 24 weeks of initial treatment [4]. With 80 % power, a 2-sided alpha of 0.2, and assuming follow-up of at least 18 months for all participants from the time of maintenance randomisation, 70 participants per arm (109 events) are required to detect a hazard ratio of 0.67, corresponding to an increase in median PFS of 6 months with Carfilzomib maintenance therapy, from the time of maintenance randomisation. Taking the required sample size for the response rate endpoint it is therefore expected that approximately 160 participants will be eligible for maintenance randomisation, providing a sufficient sample size to assess the maintenance therapy comparison, allowing approximately 10 % dropout.

### Recruitment Process

The trial is expected to take up to 36 months to complete recruitment. Participants will be recruited from NHS hospitals throughout the UK which are approved research sites within the Myeloma UK Early Phase Clinical Trial Network. Participants will be approached during standard clinic visits for management of their disease and will be provided with verbal and written details, in the form of a participant information sheet. Following information provision, participants will have as long as they need to consider participation (normally a minimum of 24 h) and will be given the opportunity to discuss the study with their family and other healthcare professionals before they are asked whether they would be willing to take part in the study.

Participants who satisfy all the study inclusion criteria and none of the exclusion criteria listed in Table 1 will be invited to provide written informed consent.

**Table 1:** MUK five study inclusion and exclusion criteria

Inclusion criteria for initial randomisation
DISEASE RELATED
- Diagnosed with symptomatic MM (according to IMWG 2003 criteria) and requiring therapy for first relapse or primary refractory disease. (Participants previously immunofixation negative who are now immunofixation positive need to demonstrate a greater than 5 g/l absolute increase in paraprotein to be eligible for inclusion).
- Participants with measurable disease as defined by one or more of the following criteria (assessed within 21 days prior to randomisation):
o Serum paraprotein ≥5 g/L (For IgA participants whose disease can only be reliably measured by serum quantitative immunoglobulin (IgA): ≥ 7.5 g/L)
o Urine Bence Jones Protein: ≥200 mg/24 h
o Serum LC assay: Involved FLC level ≥100 mg/L, provided serum FLC ratio is abnormal
DEMOGRAPHIC
- Age ≥18 years
- Life expectancy ≥6 months
- Eastern Cooperative Oncology Group (ECOG) performance status 0–2
LABORATORY
- Adequate hepatic function, with alanine transaminase (ALT) or aspartate transaminase (AST) <3 times the upper limit of normal and serum direct bilirubin ≤17 μmol/L (1 mg/100 ml) within 14 days prior to randomisation
- Absolute neutrophil count (ANC) ≥1.0 × 10^9^/L within 14 days prior to randomisation. Growth factor support is not permitted. ANC ≥ 0.8 x 10^9^/L is allowed for participants with racial neutropenia.
- Haemoglobin ≥8 g/dL (80 g/L) within 14 days prior to randomisation (participants may be receiving red blood cell [RBC] transfusions in accordance with institutional guidelines)
- Platelet count ≥75 × 10^9^/L (≥50 × 10^9^/L if myeloma involvement in the bone marrow is > 50 %) within 14 days prior to randomisation. Platelet support is not permitted
- Creatinine clearance (CrCl) ≥ 20 mL/min or plasma creatinine ≤120 μmol/L within 14 days prior to randomisation, either measured or calculated using a standard formula (e.g. Cockcroft and Gault)
ETHICAL/OTHER
- Written informed consent
- Female participants of child-bearing potential must have a negative pregnancy test prior to treatment and agree to use dual methods of contraception for the duration of the study and for 30 days following completion of study. Male participants must also agree to use a barrier method of contraception for the duration of the study and for 30 days following completion of study if sexually active with a female of child-bearing potential.
Exclusion criteria for initial randomisation
DISEASE RELATED
- Non-secretory multiple myeloma
- Extramedullary plasmacytoma (without evidence of myeloma)
- Received therapy for their first relapsed or primary refractory disease other than local radiotherapy, therapeutic plasma exchange, or dexamethasone up to a maximum of 200 mg. (Radiotherapy sufficient to alleviate or control pain or local invasion is permitted).
- Previous bortezomib therapy is permitted, providing participant was not refractory (achieved at least a PR, and no disease progression within 6 months of last dose of bortezomib)
- Previous carfilzomib therapy
CONCURRENT CONDITIONS
- Pregnant or lactating females
- Major surgery within 21 days prior to randomisation
- Acute active infection requiring treatment (systemic antibiotics, antivirals, or antifungals) within 14 days prior to randomisation
- Known human immunodeficiency virus infection (testing is not required for the trial)
- Active hepatitis B or C infection
- Unstable angina or myocardial infarction within 6 months prior to randomisation, NYHA Class III or IV heart failure, uncontrolled angina, history of severe coronary artery disease, severe uncontrolled ventricular arrhythmias, sick sinus syndrome, or electrocardiographic evidence of acute ischemia or Grade 3 conduction system abnormalities unless participant has a pacemaker, history of torsade de pointe, QTc prolongation (>450 msec), LVEF <40
- Uncontrolled hypertension or uncontrolled diabetes within 14 days prior to randomisation
- Previous or concurrent malignancy within the past 3 years with the exception of a) adequately treated basal cell carcinoma, squamous cell skin cancer, or thyroid cancer; b) carcinoma in situ of the cervix or breast; c) prostate cancer of Gleason Grade 6 or less with stable prostate-specific antigen levels; or d) cancer considered cured by surgical resection or unlikely to impact survival during the duration of the study, such as localised transitional cell carcinoma of the bladder or benign tumours of the adrenal or pancreas
- Significant neuropathy (Grades 3–4, or Grade 2 with pain) within 14 days prior to randomisation
- Participants with haemorrhagic cystitis
- Any history or known hypersensitivity to any of the study medications or excipients
- Participants undergoing active treatment for infiltrative lung disease
- Contraindication to any of the required concomitant drugs or supportive treatments, including hypersensitivity to all anticoagulation and anti-platelet options, antiviral drugs, or intolerance to hydration due to pre-existing pulmonary or cardiac impairment
- Contraindication to a programme of oral or IV hydration
- Participants with pleural effusions requiring thoracentesis or ascites requiring paracentesis within 14 days prior to randomisation
- Any other clinically significant medical disease or condition that, in the Investigator’s opinion, may interfere with protocol adherence or a participant’s ability to give informed consent

### Randomisation

Written informed consent for entry in to the trial must be obtained and eligibility must be confirmed prior to randomisation. Randomisation will be administered by telephone by the University of Leeds Clinical Trials Research Unit (CTRU), using an automated 24 h telephone system. Participants will be randomised on a 2:1 basis to either the CCD or CVD trial arm. A computer generated minimisation program that incorporates a random element will be used to ensure treatment groups are well-balanced for the following characteristics: β2 microglobulin at trial entry (<3.5, 3.5-5.5, >5.5); prior bortezomib (Velcade®) treatment (y/ n); prior ASCT (y/n); timing of first relapse or primary refractory disease (primary refractory, first relapse: < 12 months, first relapse: ≥ 12 months).

Participants on the CCD arm only, who have no evidence of progressive disease at the response assessment at the end of CCD therapy, will be randomised again to receive either carfilzomib maintenance or no maintenance treatment, provided they fulfil the eligibility criteria listed in Table 2. Participants will be randomised on a 1:1 basis to either carfilzomib maintenance or no maintenance. A computer generated minimisation program that incorporates a random element will be used to ensure treatment groups are well-balanced for the following characteristics: response category at the end of treatment with CCD (PR, MR or SD vs. VGPR or CR); prior ASCT (y/n).

**Table 2:** Maintenance randomisation inclusion and exclusion criteria

Inclusion criteria for maintenance randomisation
- Completed at least 24 weeks of CCD treatment in line with the protocol (must have received a minimum of 5 cycles and achieved a maximum response to initial therapy).
- No evidence of disease progression
- Adequate hepatic function, with ALT or AST <3 times the upper limit of normal and serum direct bilirubin ≤42.5 μmol/L (2.5 mg/100 ml) within 14 days prior to randomisation
- Absolute neutrophil count (ANC) ≥1.0 × 10^9^/L within 14 days prior to randomisation. Growth factor support received in the previous cycle of treatment is permissible.
- Haemoglobin ≥8 g/dL (80 g/L) within 14 days prior to randomisation (participants may be receiving red blood cell [RBC] transfusions in accordance with institutional guidelines)
- Platelet count ≥75 × 10^9^/L (≥50 × 10^9^/L if myeloma involvement in the bone marrow is >50 %) within 14 days prior to randomisation. Platelet support received in the previous cycle of treatment is permissible.
- Creatinine clearance (CrCl) ≥20 mL/min or plasma creatinine ≤120 μmol/L within 7 days prior to randomisation, either measured or calculated using a standard formula (eg, Cockcroft and Gault)
- Female participants of child-bearing potential must have a negative pregnancy test prior to treatment and agree to use dual methods of contraception for the duration of the study and for 30 days following completion of study. Male participants must also agree to use a barrier method of contraception for the duration of the study and for 30 days following completion of study if sexually active with a female of child-bearing potential.
Exclusion criteria for maintenance randomisation
- Uncontrolled hypertension or uncontrolled diabetes
- Any other clinically significant medical disease or condition that, in the Investigator’s opinion, may interfere with protocol adherence
- Pregnant or lactating females
- Significant neuropathy (Grades 3–4, or Grade 2 with pain)

### Intervention

Participants randomised to CVD will receive the following regimen: bortezomib subcutaneous 1.3 mg /m^2^ (days 1, 4, 8 and 11), cyclophosphamide oral 500 mg (days 1, 8 and 15), dexamethasone oral 40 mg (days 1, 8 and 15). The cycle is repeated every 21 days. Response should be assessed at the end of each cycle and, in the absence of disease progression or intolerance, participants should receive 8 cycles of treatment. Participants randomised to CCD will receive the following regimen: carfilzomib IV 20 mg/m^2^(cycle 1, days 1 and 2 only) / 36 mg/m^2^ (cycle 1, days 8, 9, 15 and 16, and cycle 2 onwards, days 1, 2, 8, 9, 15 and 16), cyclophosphamide oral 500 mg (days 1, 8 and 15), dexamethasone oral 40 mg (days 1, 8, 15 and 22). The cycle is repeated every 28 days. Response should be assessed at the end of each cycle and, in the absence of disease progression or intolerance, participants should receive 6 cycles of treatment.

Participants in the CCD arm, who are randomised to receive carfilzomib maintenance, will receive carfilzomib IV 36 mg/m^2^ (days 1, 2, 15 and 16) for 6 months, after which the frequency of dosing will be reduced to carfilzomib IV 36 mg/m^2^ (days 1 and 2) for a further 12 months.

Each drug may be reduced due to toxicity. If no resolution of toxicity is seen after adjustment to the lowest dose level, treatment will be discontinued.

### Trial assessments

Baseline investigations are to be performed within 14 days prior to randomisation, after written informed consent has been obtained. The required baseline assessments include a physical examination, medical history, ECOG performance status and ISS stage, as well as haematology, biochemistry, radiological and bone marrow sample assessments.

Assessments during treatment, including a physical examination, safety and laboratory tests will be performed at multiple time-points during cycles 1 – 6 for the CCD regimen and cycles 1 – 8 for the CVD regimen. Response assessments will be carried out at the end of treatment phase (6 months in each arm), according to International Myeloma Working Group (IMWG) criteria, and will include bone marrow examination for MRD using multi-parameter flow cytometry.

During maintenance in the CCD arm, assessments will be performed on days 1, 2, 15 and 16 of each cycle (or days 1 and 2 if administering the reduced schedule) and will include a physical examination and laboratory tests. Assessments at 6 and 12 months will also include bone marrow sampling for MRD assessment.

Assessments at the end of initial and maintenance treatment include a physical examination, laboratory tests and a bone marrow aspirate (post-initial treatment). Follow-up will be performed 4-weekly until disease progression, and will involve a physical examination and laboratory tests.

### Outcome measures

The study has two co-primary endpoints, the proportion of participants achieving at least VGPR 24 weeks post initial randomisation and progression-free survival. A key secondary endpoint is the proportion of participants experiencing ≥ grade 3 neuropathy or ≥ grade 2 neuropathy with pain during the initial treatment period (8 cycles of CVD or 6 cycles of CCD). All secondary endpoints are listed in Table 3.

**Table 3:** MUK five secondary endpoint analysis

Secondary endpoint	Main comparison(s)	Analysis method(s)
Key secondary: proportion of participants experiencing ≥ grade 3 neuropathy or ≥ grade 2 neuropathy with pain during initial treatment	(a) vs. (b)	[S] Logistic regression
(i) Total time spent at each grade of toxicity for ≥ grade 3 neuropathy or ≥ grade 2 neuropathy with pain during initial treatment	(a) vs. (b)	[NFT] Descriptive summaries
(ii) Complete response rate 24 weeks post initial randomisation	(a) vs. (b)	[NI] Logistic regression
(iii) Overall response rate 24 weeks post initial randomisation	(a) vs. (b)	[NI] Logistic regression
(iv) Proportion of participants achieving MRD negative disease at the end of initial treatment	(a) vs. (b)	[NI] Logistic regression
(v) MRD status of participants at 6 and 12 months following second randomisation and alteration in MRD status	(c) vs. (d)	[S] Logistic regression
(vi) PFS by MRD status at the end of initial treatment	MRD -ve vs. MRD + ve	[S] Kaplan-Meier & log-rank test, Cox PH
(vii) Overall response rate within 12 months of initial randomisation	(c) vs. (d), (a) vs. (e)	[S] Logistic regression
(viii) Maximum response within 12 months of initial randomisation	(c) vs. (d), (a) vs. (e)	[NFT] Descriptive summaries
(ix) Maximum response overall	(c) vs. (d), (a) vs. (e)	[NFT] Descriptive summaries
(x) Time to maximum response	(c) vs. (d), (a) vs. (e)	[S] Kaplan-Meier & log-rank test, Cox PH
(xi) Duration of response	(c) vs. (d), (a) vs. (e)	[S] Kaplan-Meier & log-rank test, Cox PH
(xii) Overall survival	(c) vs. (d), (a) vs. (e)	[S] Kaplan-Meier & log-rank test, Cox PH
(xiii) Time to next treatment	(c) vs. (d), (a) vs. (e)	[NFT] Kaplan-Meier; Cumulative Incidence Functions (competing risks)
(xiv) Toxicity overall, and per cycle of treatment (initial and maintenance treatment)	(a) vs. (b), (c) vs. (d)	[NFT] Descriptive summaries, graded by Common Terminology Criteria for Adverse Events V4.0
(xv) Treatment compliance	(a) vs. (b), (c) vs. (d)	[NFT] Descriptive summaries
(xvi) Safety	(a) vs. (b), (c) vs. (d)	[NFT] Descriptive summaries

*NI* non-inferiority, *S* superiority, *NFT* no formal statistical testing, *Cox PH* Cox’s proportional hazards model

### Statistical methods and analysis

Participants will be grouped as follows for analysis:

- All participants randomised to the CVD arm in the initial randomisation
- All participants randomised to the CCD arm in the initial randomisation
- Participants in the CCD arm who were, at the maintenance randomisation, randomised to receive no maintenance therapy
- Participants in the CCD arm who were, at the maintenance randomisation, randomised to receive maintenance therapy
- Participants in the CCD arm who do not receive maintenance therapy. This includes participants who are, at the maintenance randomisation, randomised to receive no maintenance therapy, plus those who do not undergo maintenance randomisation either because of not being eligible or not being willing. N.B. in order to avoid bias due to the imbalanced randomisation schedule, analyses may include all participants in the CCD arm, adjusting for the effect of maintenance therapy as appropriate to provide a comparator group representing CCD arm participants who do not receive maintenance therapy.

#### Primary endpoint analysis

The primary endpoint analysis will include all participants who have received at least one full cycle of their allocated chemotherapy. Participants who received less than one full cycle, defined as missing more than 2 doses of either Carfilzomib or bortezomib in cycle 1 and then stopping trial treatment, will be monitored and analysed separately as applicable. A non-inferiority analysis of the proportion of participants achieving at least VGPR 24 weeks post initial randomisation will compare groups (a) and (b) in terms of the proportion of participants achieving ≥ VGPR 24 weeks post randomisation, with a null hypothesis of inferiority of the CCD arm as compared to the CVD arm, and an alternative hypothesis of non-inferiority of CCD, using a pre-specified non-inferiority margin of −5 %. Further analysis using logistic regression will adjust for the minimisation factors (β2 microglobulin at trial entry, prior bortezomib treatment, prior autologous stem cell transplant, and timing or first relapse or primary refractory disease). Treatment and covariate estimates with corresponding standard errors, odds ratios, 90 and 95 % confidence intervals and *p*-values will be presented.

The main comparison for PFS will be a superiority analysis of groups (c) and (d). PFS curves will be calculated using the Kaplan-Meier method and the median progression-free survival estimates and progression-free survival estimates at 12 and 24 months with corresponding 80 % and 95 % confidence intervals will be presented by treatment group. A log-rank test, stratifying for the minimisation factors, will be used to compare PFS between treatment groups. Cox’s proportional hazards model (if appropriate), adjusting for the minimisation factors, will also be used to compare PFS between the treatment groups. Treatment and covariate estimates, standard errors, hazard ratios, 80 % and 95 % confidence intervals, as well as *p*-values will be presented. An exploratory non-inferiority comparison will also be performed to compare groups (a) and (e). If the main comparison between (c) and (d) shows no evidence of improved PFS for either treatment group, the superiority analyses described above will also be performed for (a) vs (b).

Analysis of response and PFS will be performed using the data recorded on the case report form, which will be centrally reviewed for quality assurance.

#### Secondary endpoint analysis

The main comparisons and analysis methods for each secondary endpoint are given in Table 3. Short term endpoints (i.e. those evaluable at 24 weeks post randomisation / the end of initial treatment) generally compare groups (a) and (b). Longer term endpoints generally compare groups (c) and (d), and (a) and (e). The majority of endpoints will be analysed according to the analysis population, including all participants who receive at least one cycle of their allocated chemotherapy, as with the primary endpoint analysis. The safety population will include all participants who receive at least one dose of any trial treatment and will be used for the toxicity and safety endpoints. Treatment compliance will be produced for all participants according to the trial pathways. Exploratory analyses to investigate the association between clinical outcomes (ORR and PFS) and genetic subgroups will also be performed. Flow cytometry for MRD detection will be performed as reported by Rawstron et al. [18], with a 0.01 % limit of detection, assessing 500 000 cells incubated with 6-color antibody combinations including CD138/CD38/CD45/CD19 with CD56/CD27 in all cases and CD81/CD117 in some cases, as required. All analyses are pre-defined in a statistical analysis plan prior to any analysis being undertaken.

#### Frequency of analyses

A Data Monitoring and Ethics Committee (DMEC) will be set up to independently review data on safety, activity, protocol adherence and recruitment. This committee, in light of this data, and any advice and evidence they wish to request, will if necessary report to the Trial Steering Committee (TSC) if there are any concerns regarding the activity or safety of the trial treatments.

An interim analysis for inferiority will be performed once half the number of patients has been recruited. This interim analysis will assess for inferiority of CCD, as compared to CVD, in terms of the response co-primary endpoint. If, at the time of this interim analysis, CCD is found to be significantly inferior as compared to CVD, the DMEC will report to the TSC with a recommendation of early trial closure. The analysis will be detailed in a DMEC Interim Statistical Analysis Plan.

Final analysis is planned to take place in two stages. Stage 1 of the analysis is planned to take place after all participants have completed the 24 weeks of initial treatment and will include the short term endpoints relating to the initial treatment period or 24 weeks post initial randomisation time-point. Stage 2 of the final analysis is planned to take place when all participants have completed their full follow-up. This analysis will consider all long-term endpoints not included in stage 1 of the analyses.

## Discussion

Multiple myeloma is the 17th most common cancer in the UK, accounting for around 1 % of all new cancer cases. Myeloma incidence rates have increased overall in Great Britain since mid-1970s [1]. The majority of patients will enter a plateau phase lasting some 3–5 years, with therapies based on thalidomide or alkylating agents. Patients will relapse and require further anti-myeloma therapy. The use of bortezomib and lenalidomide has revolutionised the treatment of relapsed disease with both drugs being shown to improve response rates, progression-free and overall survival, when compared to dexamethasone in randomised clinical trials in relapsed participants [2].

The triple combination of bortezomib, dexamethasone and an alkylating agent, such as cyclophosphamide (CVD), is an effective regimen at relapse. The development of carfilzomib offers the opportunity to further explore the anti-tumour efficacy of proteasome inhibition. In phase I and phase II clinical studies, carfilzomib demonstrated robust and durable efficacy and acceptable safety and tolerability profile in participants with relapse and/or refractory multiple myeloma [19]. Based on the available evidence, it is important and timely to obtain data on the activity, toxicity and tolerability of this drug. In contrast to ongoing phase III trials such as ASPIRE, FOCUS, ENDEAVOR and CLARION [19], this phase II trial has a unique subset of participants diagnosed with multiple myeloma at first relapse or refractory to no more than 1 line of treatment. The MUK five trial will also evaluate the utility of maintenance with carfilzomib for up to 18 months and investigate MRD status to provide information on depth of response and the prognostic impact thereof.

The findings of this trial will inform healthcare professionals, patients, and their caregivers about the activity and safety profile of CCD in this setting.

### Ethics

The trial has received national research ethics approval from the NHS National Research Ethics Service London, Fulham (REC Number: 12/LO/1078)

### Consent to participate

All patients provide written informed consent prior to randomisation in to the trial.

### Consent to publish

Not applicable

### Availability of data and materials

Not applicable – this is a protocol paper outlining the study being conducted. All data supporting the development of the study is outlined in the manuscript.

## Abbreviations

ALT: alanine transaminase
ANC: absolute neutrophil count
ASCT: autologous stem cell transplant
AST: aspartate transaminase
CR: complete response
DMEC: data monitoring and ethics committee
IV: intravenous
IMWG: international myeloma working group
MR: minimal response
MRD: minimal residual disease
MUK: myeloma UK
ORR: overall response rate
PFS: progression-free survival
PR: partial response
SC: subcutaneous
SD: stable disease
VGPR: very good partial response
